# Diabetes Mellitus Secondary to Cushing’s Disease

**DOI:** 10.3389/fendo.2018.00284

**Published:** 2018-06-05

**Authors:** Mattia Barbot, Filippo Ceccato, Carla Scaroni

**Affiliations:** Endocrinology Unit, Department of Medicine DIMED, University of Padua, Padua, Italy

**Keywords:** Cushing’s disease, diabetes, glucocorticoids, insulin resistance, cortisol-lowering medication

## Abstract

Associated with important comorbidities that significantly reduce patients’ overall wellbeing and life expectancy, Cushing’s disease (CD) is the most common cause of endogenous hypercortisolism. Glucocorticoid excess can lead to diabetes, and although its prevalence is probably underestimated, up to 50% of patients with CD have varying degrees of altered glucose metabolism. Fasting glycemia may nevertheless be normal in some patients in whom glucocorticoid excess leads primarily to higher postprandial glucose levels. An oral glucose tolerance test should thus be performed in all CD patients to identify glucose metabolism abnormalities. Since diabetes mellitus (DM) is a consequence of cortisol excess, treating CD also serves to alleviate impaired glucose metabolism. Although transsphenoidal pituitary surgery remains the first-line treatment for CD, it is not always effective and other treatment strategies may be necessary. This work examines the main features of DM secondary to CD and focuses on antidiabetic drugs and how cortisol-lowering medication affects glucose metabolism.

## Introduction

Cushing’s disease (CD) is a rare pathology characterized by uncontrolled ACTH secretion from a pituitary adrenocorticotroph adenoma that leads to an increase in cortisol production by the adrenal glands (1). It is a serious condition characterized by metabolic derangements that may include visceral adiposity, hepatic steatosis, dyslipidaemia, and diabetes mellitus (DM) (2, 3). The prevalence of DM in CD patients is thought to fall between 20 and 45%, although the figure may be underestimated, as an oral glucose tolerance test may not be performed when a patient’s fasting glycemia is normal (4). Approximately 10–30% of patients have impaired glucose tolerance, and the overall prevalence of glucose metabolism impairments reaches nearly 70% of cases (4). No gender-related differences in prevalence have been noted (5). Generally speaking, the severity of hypercortisolism is correlated with insulin resistance and DM (6), although the correlation has not always been confirmed (7, 8). This discrepancy might depend on the wide inter-individual susceptibility to glucocorticoids. Age, genetic predisposition, and lifestyle variables combined with the duration and degree of hypercortisolism may all strongly contribute to glucose tolerance impairment in CD patients (9).

## Pathophysiology

**Figure 1:**
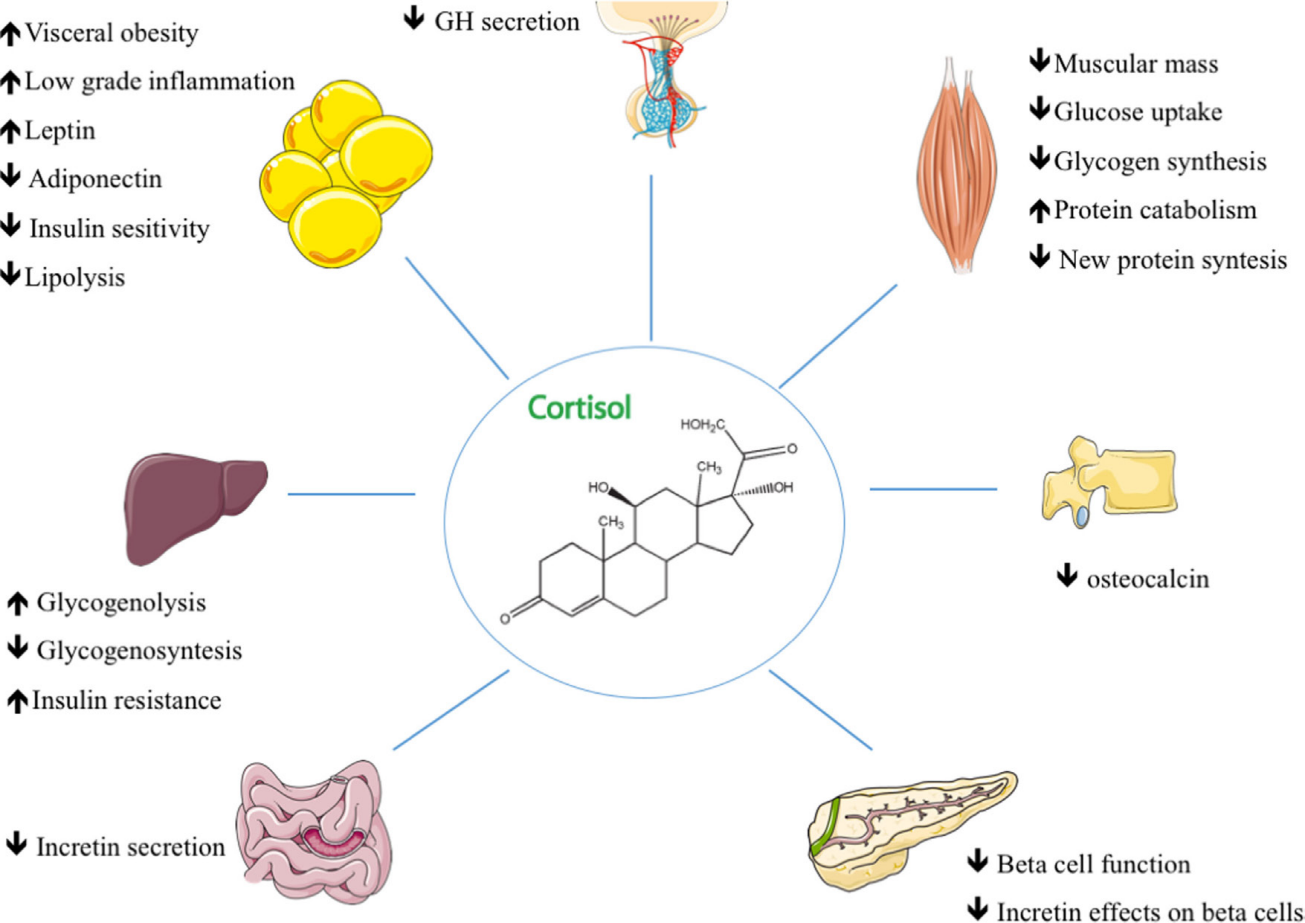
The main mechanisms of action in glucocorticoid-induced diabetes and their effects on target tissues in Cushing’s disease.

Cortisol, which is a steroid hormone, regulates a wide range of body processes, but it displays its main effect after food intake. It seems to contribute to glucose intolerance and to reduce insulin sensitivity (10). In the liver, chronic hypercortisolism impairs fasting and postprandial glucose (Figure 1). Cortisol exacerbates gluconeogenesis and hepatic glucose output through both direct and indirect effects (4). It regulates glucocorticoid-responsive target genes by upregulating key gluconeogenic enzymes such as phosphoenolpyruvate carboxykinase and glucose-6-phosphatase (4). Chronic glucocorticoid exposure also induces selective insulin resistance that impedes the inhibitory effect of insulin on hepatic glucose output (11). Skeletal muscles account for 70–80% of the body’s use of glucose. CD increases the rate of proteolysis and of muscle mass loss (12) with consequent decreased muscle insulin responsiveness and impaired glucose uptake (13). Abdominal obesity is closely associated with metabolic syndrome and CD; adipocyte-derived lipids in visceral adipose tissue can contribute to the onset of peripheral insulin resistance and DM by promoting altered insulin signaling in adipocytes, increased lipolysis, aberrant adipokine secretion, and low-grade inflammation (4). Although human studies have uncovered normal incretin secretion and a reduction in its insulin-releasing effects on β-cells, the incretin system seems to be affected, in animals, by hypercortisolemia since a blunted GLP-1 secretion has been observed in response to glucocorticoid administration (14, 15). Cortisol is necessary for the normal development of the adrenal medulla that is specialized in the synthesis, storage, and secretion of catecholamines from chromaffin cells; its excess enhances the adrenergic-mediated increase in gluconeogenesis (16). Cortisol excess also has an important impact on GH/IGF-1 leading to an increase in visceral fat and insulin resistance (17, 18). Bone has also been found to be involved in glucose homeostasis (19); prolonged glucocorticoid exposure causes a reduction in circulating osteocalcin which in turn can enhance insulin resistance (20). Cortisol’s biological effects can be modulated in target tissues by the activity of 11β-hydroxysteroid dehydrogenase (11βHSD) enzymes; isoform 1 converts cortisone into cortisol, amplifying its action in the liver and adipose tissue where it is primarily expressed (21). The overexpression of 11βHSD1 in adipose tissue is correlated with the development of insulin resistance and obesity (22). Finally, glucocorticoid receptor polymorphisms may also play a role in the development of metabolic complications (23). The A3669G polymorphism has been found to play a protective role in CD (24) by producing glucocorticoid resistance that increases the expression and stabilization of the dominant-negative GR-β splice variant (24, 25).

## Therapy

As diabetes is a consequence of cortisol excess, treating the underlying disease is unquestionably indispensable in these patients. Despite increasing interest in medical therapy, the first-line approach still remains transsphenoidal surgery (26) which provides, when it is performed by an experienced neurosurgeon, a mean remission rate of 77.8% (3, 27). Normalized cortisol levels after surgery is generally followed by an improvement in the patient’s glucose metabolism, but insulin resistance and cardiovascular risks may persist (28), particularly in patients with a genetic predisposition and/or persistent visceral adiposity (9). This means that specific, although usually at lower doses, hypoglycemic treatment may be required even after remission following surgery (4).

## Antidiabetic Treatment

Although correcting cortisol hypersecretion leads to an improvement in glucose homeostasis, patients with CD and DM need to achieve glycemic control regardless of normalized cortisol levels. Treating diabetes in patients with CD does not essentially differ from treating it in ordinary circumstances (29). There are nevertheless some pathophysiological features that should be considered. Physical activity can be recommended in CD patients although resistance exercises should be limited in patients with glucocorticoid-induced myopathy (30). Metformin remains the first-line therapy of hyperglycemia in CD (31). While metformin lowers fasting plasma glucose concentrations by reducing hepatic glucose production (32) without causing hypoglycemia or weight gain (33), it may have undesired gastrointestinal side effects, that can be worsened by the concomitant use of pasireotide (34). Extended-release formulas can improve gastrointestinal tolerability (35). Acarbose inhibits the enzymatic cleavage of complex carbohydrates, delaying their absorption and thereby reducing postprandial glycemic excursions (36). Although it does not seem to have negative effects on body weight, it often produces gastrointestinal side effects (37). Peroxisome proliferator-activated receptor-γ (PPARγ) agonists interact with the nuclear receptor PPARγ ameliorating insulin resistance and improving hepatic and muscle insulin sensitivity (38). They may also potentiate the effects of metformin (39) on glycemic control and trigger anti-proliferative effects on tumor cells. Pioglitazone treatment has not, however, been found to reduce ACTH or cortisol levels in CD (40, 41) and may cause weight gain and/or edema which may worsen the CD patient’s clinical condition (4, 42, 43).

Sulfonylureas and glinides stimulate insulin secretion in a glucose-independent manner triggering both the immediate and sustained release of insulin from intracellular granules (44). They are rarely used independently, but are mainly recommended for short-term periods to manage postprandial glycemia (45). Other drugs that produce good postprandial glucose control are dipeptidyl peptidase-4 inhibitors, which do not seem to affect weight (46), and since they are well tolerated, they are usually associated with metformin in cases of treatment intensification (47). GLP-1 receptor agonists act by enhancing glucose-induced biosynthesis and insulin secretion, inhibiting glucagon secretion, delaying gastric emptying, and reducing appetite (48, 49). Other positive effects such as weight loss and blood pressure reduction have also been noted (50). Incretin-based therapy may be the best treatment option for CD given its rapid action and positive effect on postprandial glycemia (51). Sodium-glucose co-transporter 2 inhibitors are a new class of diabetic drugs (52) that cause a reduction in body weight and blood pressure (53). In the light of reports linking them to a higher risk of urinary and genital infections (54), the decision to use them should be carefully evaluated in CD patients who show a high risk of infections and systemic dissemination (55). Another possible, although rare, side effect that has been reported in diabetic patients is the development of euglycaemic ketoacidosis probably caused by an increase in glucagon levels and consequently enhanced ketogenesis (56). But given their proven capacity to improve glycemic control and their association to a low rate of cardiovascular events (57), their use should be considered in appropriate cases. Insulin may become necessary in patients in whom uncontrolled diabetes persists (29). Basal-bolus insulin therapy including basal, prandial, and a supplemental correction-factor insulin is considered the most flexible option for patients with hypercortisolism (58).

## Pasireotide

Pasireotide, a multi-somatostatin receptor ligand that is able to bind four of the five known SSTR subtypes (SSTRs1–3 and SSTR5) with a 40-fold greater binding affinity for SSTR5 with respect to octreotide (59, 60), was the first agent to be approved for the treatment of adult CD patients. Pasireotide has the same security profile of first-generation somatostatin analogs although it is associated with a relatively high incidence of hyperglycemia (61, 62) due to the expression of SSTR5 even in pancreatic β-cells (63). When the pathophysiology of pasireotide-induced hyperglycemia was investigated in healthy volunteers (64, 65), short-term administration was found to be followed by an increase in blood glucose levels associated with reduced insulin secretion (66) and no significant alteration in glucagon output (60). In addition, pasireotide can potentially reduce the secretion of other pituitary hormones (67). Although a decrease in the counterregulatory action of GH can reduce insulin resistance and hepatic gluconeogenesis, its deficiency can further increase metabolic complications as a result of body composition changes (68).

Clinical practice has shown that glucose alterations are more common and serious at the time therapy is begun but tend to stabilize over time. In some cases, an improvement in glucose control has been reported following long-term treatment, probably as a result of an improvement in insulin resistance (69). Interestingly, pasireotide-induced hyperglycemia seems to be independent of the doses that are assumed (65). Besides reducing insulin secretion, pasireotide also has a significant inhibitory effect on GLP-1 and on glucose-dependent insulinotropic peptide secretion (65).

In a phase II, proof-of-concept, open-label multicenter study, 39 patients with CD were treated with 600 µg twice daily pasireotide for 15 days. There was a significant reduction in urinary free cortisol (UFC) values in 2/3 of the patients and complete normalization in 17%. Hyperglycemia was found in 14 patients; 5 of whom with a history of DM or impaired fasting glucose. Hyperglycemia, which was mild in most of the cases, was managed with diet and oral hypoglycemic agents (61).

The effectiveness of this treatment was confirmed by a phase III clinical trial in which 15 and 26% of patients achieved UFC normalization after 6 months of treatment with, respectively, 600 or 900 µg of pasireotide twice daily (62). Hyperglycemia was reported in up to 73% of cases; the rate was significantly higher than that observed for first-generation SST analogs. Treatment needed to be suspended because of uncontrolled DM in 6% of the patients (62). A rise in HbA1c levels from 5.8 to 7.3% was recorded after 12 months of pasireotide. Blood glucose and HbA1c levels increased rapidly after pasireotide treatment was begun despite a decline in cortisol levels (62).

A recent phase III study examining a long-acting formulation of pasireotide confirmed the advantage of its convenient once-monthly administration schedule. In addition, long-acting pasireotide normalized UFC in 40% of the patients and its safety profile was found to be similar to that of the subcutaneous formulation (67). Mean fasting plasma glucose and HbA1c concentrations increased within 1–2 months and antidiabetic medication needed to be initiated or adjusted in approximately in 50% of cases (67). It is therefore crucial to carefully check blood glucose concentrations in patients initiating pasireotide and to begin glucose-lowering therapy promptly whenever it proves necessary (47).

## Dopamine Agonist

Since dopamine receptor agonist is able to reduce hypothalamic stimulation that increases during liver gluconeogenesis, lipid synthesis, and insulin resistance (70, 71) bromocriptine has been used to treat metabolic disorders associated with insulin resistance and obesity (72). When cabergoline, a potent dopamine agonist was tested in patients with type 2 diabetes, its ability to reduce both fasting glycemia and HbA1c was confirmed (71). Cabergoline may also improve glycemic control through weight loss, although this effect has not been consistently found by all studies (73). Since dopamine D2 receptor expression was found in almost 80% of adenocorticotroph cells, cabergoline could be an efficacious treatment for CD (74); some studies have reported that cabergoline is effective in controlling hypercortisolism in 30–45% of patients (75–77). Diabetes and glucose intolerance ameliorated in 60 and 46%, respectively, irrespective of cortisol levels (75). A recent large retrospective multicenter study on 62 patients confirmed already published results reporting long-term cortisol normalization in 20–25% of cases and a significant improvement in glycemic control in 40% of cases (78).

## Retinoic Acid (RA)

After it was shown to be involved in reducing ACTH secretion and tumor growth in *in vitro* and animal models *via* inhibition of POMC expression in corticotroph tumors, RA, a nuclear receptor ligand, has been considered another potential option for CD treatment (79). The first clinical study examining its effect on seven CD patients demonstrated a UFC reduction ≥50% in five out of seven patients after 6 months of treatment and a complete response in three of the cases. There was a significant improvement in glycemia and HbA1c (decreased by 0.4–1.2%) in all five patients with DM at baseline (80). The 13-cis isomer of RA was recently examined by an open-label trial; UFC normalization was reached in 4 out of the 16 patients at 12 months, with up to a 52% reduction in UFC in the rest. There was also an overall significant reduction in fasting glycemia (81). RA’s ameliorative effect on glucose metabolism can be attributed to both its cortisol-lowering action and its direct effects. In fact, retinol active metabolites are able to stimulate insulin secretion, enhance mRNA expression of glucose transporter GLUT2, and promote lipolysis in adipocytes by activating PPARγ (82, 83) (Table 1).

**Table 1:** Medications available to treat Cushing’s disease and their effects on glucose metabolism.

Drug	Mechanisms of action	Usual dose	Hormonal control	Overall effect on glucose metabolism	Effects on glucose metabolism
Cabergoline (75–77)	Acts through D2R receptors express on adenocorticotroph	0.5–7 mg/week oral	25–40%	⇑	↓ Insulin resistance ↓ Gluconeogenesis

Ketoconazole (87–90)	Cholesterol side-chain cleavage complex, 17,20-lyase, 11β-hydroxylase and 17α-hydroxylase inhibitor	200–1,200 mg/day 2–3 times/day, oral	~50%	⇑	↓ Cortisol levels

Osilodrostat (97) (LCI699)	11β-hydroxylase and aldosterone synthase inhibitor	4–60 mg/day 2 times/day, oral	~90%	⇑	↓ Cortisol levels

Metyrapone (93–95)	11β-hydroxylase inhibitor	0.5–6 g/day 3–4 times/day, oral	45–75%	⇑	↓ Cortisol levels

Mifepristone (99, 100)	Glucocorticoid receptor antagonist	300–1,200 mg/day Once daily, oral	Na	⇑	↓ Cortisol effects on target tissues

Mitotane (86, 98)	Cholesterol side-chain cleavage complex, 11β-hydroxylase, 18-hydroxylase and 3β-hydroxysteroid-dehydrogenase inhibitor + adrenolytic action	2–5 g/day 2–3 times/day, oral	~70%	⇑	↓ Cortisol levels

Retinoic acid (80–83)	Reduces ACTH production through inhibition of AP-1 and Nur77/Nurrl transcriptional activities	10–80 mg/day 1–3 times/day, oral	20–50%	⇑	↓ Cortisol levels ↓ Insulin resistance ↑ Insulin secretion

Pasireotide (61, 62, 65)	Somatostatin multi-ligand with particularly high SSTR5	300–1,800 μg/day Twice a day, sc	20–50%	⇓⇓	↓ Insulin production ↓ Incretins secretion

## Ketoconazole

Ketoconazole is an imidazole derivative that reduces adrenal steroid production by inhibiting numerous steroidogenic enzymes (84, 85). Although it has been used for decades, no perspective studies are as yet available (86). At doses of 200–1,200 mg/day, it is able to improve glucose metabolism in CD patients (87–90). Ketoconazole enantiomer (DIO-902) has been found to be effective at lowering HbA1c, fasting glucose, total cholesterol levels, and LDL cholesterol levels (91).

A French study retrospectively assessed 38 CD patients receiving ketoconazole (200–1,200 mg/day) for a median of 23 months; the 5 patients who had DM all achieved cortisol regulation and an improvement in metabolic control (88). Another retrospective study examining 62 CD patients receiving steroidogenesis inhibitors as pre-surgical treatment (ketoconazole, metyrapone, or their combination) reported that HbA1C levels fell in those patients whose cortisol levels were entirely or partially controlled, but it became necessary to gradually increase insulin or prescribe oral antidiabetic drugs for the non-controlled patients (89).

A large retrospective multicenter study by Castinetti et al. reviewing data on 200 CD patients treated with ketoconazole monotherapy; at baseline, 31.8% of the patients had DM. Glycemic control improved in more than half of the diabetic patients after ketoconazole therapy (90).

## Metyrapone

Metyrapone inhibits the final step in cortisol synthesis, namely the conversion of 11-deoxycortisol into cortisol by 11β-hydroxylase (86). Due to its rapid action, it is particularly suitable to achieve cortisol control within a few days’ time and thus to improve glucose metabolism over a short time period (92). Jeffcoate et al. evaluated the efficacy of metyrapone at doses ranging from 500 to 4,000 mg/day in 13 CD patients after a mean of 21 months. Of the seven who had an abnormal glucose tolerance at the baseline, five showed improvement after 3 months of treatment (93). Another study reported a significant improvement in glucose metabolism in more than 80% of CD patients (94). Daniel et al. recently conducted a large retrospective study on metyrapone in 195 CD patients, 35% of whom were diabetic. The agent’s effects on glucose metabolism were not analyzed by the study, but hypoglycemia was reported in three patients taking antidiabetic drugs following an improvement in hypercortisolism (95).

## Osilodrostat

Osilodrostat (LCI699), an adrenal steriodogenesis inhibitor developed for the treatment of CD, is currently undergoing investigation. Its mechanism of action is similar to that of metyrapone, it is a potent inhibitor of 11β-hydroxylase and aldosterone synthase (86). A 10-week, proof-of-concept study examining 12 CD patients receiving osilodrostat, reported no important changes in insulin levels, although a nearly significant decrease in HbA1c was noted (96).

A subsequent 22-week, multicenter, prospective, open-label, phase II study examined the agent’s effect on 19 CD patients. Although the proportion of responders reached 90% as far as glucose metabolism was concerned, fasting plasma glucose and HbA1c levels fell from baseline to week 22; the greatest reduction was noted in patients with previous DM (97).

## Mitotane

Given its strong adrenolytic effect, mitotane has been widely used as an adjuvant treatment for adrenal carcinoma (26). Since its activity is long lasting, the medication is also prescribed to patients with CD (86). Baudry et al. retrospectively reviewed the clinical charts of 76 patients from a single center who were treated with mitotane. Remission with a statistically significant improvement in both fasting and postprandial serum glucose levels was achieved in 48 (72%) of those receiving the drug for at least 6 months (98).

## Mifepristone

Mifepristone is a glucocorticoid receptor antagonist that was approved by the U.S. Food and Drug Administration in 2012 for the treatment of hyperglycemia in CS patients who are not candidates for surgery (4). An open-label, multicenter, prospective, 6-month study was conducted on 50 patients with endogenous CS (43 CD) who were refractory to other therapies. Study results showed that 29 had DM or IGT. After 24 weeks of mifepristone treatment, fasting plasma glucose and HbA1c decreased, respectively, from 8.3 ± 4.1 to 5.8 ± 2.1 mmol/L and from 7.43 ± 1.52 to 6.29 ± 0.99%, leading to a reduction in the number of antidiabetic medications the patients were taking (99). A large percentage of the patients showed improved insulin resistance, with the greatest amelioration taking place during the first 6 weeks of treatment, suggesting that the early rapid improvement was linked to the direct effects of glucocorticoid blockade, while the later one depended on weight loss (100).

## Combination Drug Therapy

As no single drug has shown complete efficacy, combining drugs with additive, synergistic actions, is a strategy that has been used to increase the possibility of controlling hypercortisolism using lower doses (101) and more effectively managing glucose metabolism. A small prospective trial examining 14 patients with CD found that combining cabergoline and ketoconazole was more efficacious than using either of the two drugs alone, not only with regard to hormonal control but also as far as glucose metabolism was concerned (102). Other combinations such as the association of pasireotide-cabergoline and ketoconazole have also been utilized. One small trial found a complete response in 88% of patients receiving pasireotide subcutaneously and cabergoline. Glucose homeostasis alterations, which have been linked to pasireotide treatment, were, nevertheless, common (glycated hemoglobin level, 5.8 ± 0.2 to 6.7 ± 0.3%). Since another drug was being used, it was possible to use lower doses of pasireotide and thus to reduce its detrimental effect on glycemia (103). A metyrapone, ketoconazole, and mitotane combination was utilized in 11 patients with severe CD (4 cases of CD) as an alternative to rescue adrenalectomy. All the patients showed a rapid clinical improvement; five of the eight diabetic patients showed improved glycemic control (104).

## Conclusion

Early diagnosis can reduce disease-related complications and improve life expectancy in CD patients, and DM is one of its most frequent although underestimated complications. Appropriate treatment is based on antidiabetic medication and, first and foremost, treating the underlying disease. Transsphenoidal surgery remains the most effective treatment to control both cortisol and glucose metabolism as it can guarantee long-term remission in a high percentage of patients, but other options need to be considered when it is ineffective or unfeasible. With the exception of pasireotide, all cortisol-lowering medications have been shown to be effective in reducing to some degree the severity of hyperglycemia. Due to its action on peripheral insulin sensitivity, which is the primary mechanism responsible for glucose intolerance in CD, metformin represents the mainstay of antidiabetic treatment. When treatment intensification becomes necessary, incretin-based therapies may represent a useful option. Beyond glucocorticoid excess, other factors implicated in DM development such as age, genetic predisposition, and lifestyle variables combined with the duration and degree of hypercortisolism, may contribute to impaired glucose tolerance.

## Author Contributions

MB: literature revision and drafting of the article. FC: drafting of the article. CS: critical revision of the article and final approval.

## Conflict of Interest Statement

The authors declare that the research was conducted in the absence of any commercial or financial relationships that could be construed as a potential conflict of interest. The handling Editor declared a past co-authorship with the authors.
